# Supplementary material for: Mechanisms governing the pioneering and redistribution capabilities of the non-classical pioneer PU.1
Source: Nat Commun. 2020 Jan 21;11:402. doi: 10.1038/s41467-019-13960-2 (PMC6972792; doi:10.1038/s41467-019-13960-2)
Supplement: Supplementary file 7 — Source data [file 41467_2019_13960_MOESM7_ESM.zip › Source_Data/FigureS2/FigureS2B_MotifScanOutput/KmeansCluster1/homerResults/motif23.similar.html]

motif23

## Information for motif23

A
C
G
T
G
A
T
C
G
A
T
C
A
C
G
T
A
G
T
C
G
T
A
C
C
G
A
T
A
G
T
C
A
C
G
T
A
C
G
T
G
T
A
C
A
G
T
C
C
G
A
T
C
A
T
G
  
Reverse Opposite:  

G
T
A
C
C
G
T
A
A
C
T
G
C
A
T
G
G
T
C
A
C
G
T
A
T
C
A
G
C
G
T
A
C
A
T
G
T
C
A
G
C
G
T
A
C
T
A
G
C
T
A
G
T
G
C
A
  

|  |  |
| --- | --- |
| p-value: | 1e-33 |
| log p-value: | -7.767e+01 |
| Information Content per bp: | 1.688 |
| Number of Target Sequences with motif | 173.0 |
| Percentage of Target Sequences with motif | 3.40% |
| Number of Background Sequences with motif | 400.1 |
| Percentage of Background Sequences with motif | 1.01% |
| Average Position of motif in Targets | 123.8 +/- 50.8bp |
| Average Position of motif in Background | 85.7 +/- 80.6bp |
| Strand Bias (log2 ratio + to - strand density) | 0.4 |
| Multiplicity (# of sites on avg that occur together) | 1.00 |
| Motif File: | file (matrix) reverse opposite |

### Similar de novo motifs found

|  |  |  |  |  |  |  |  |
| --- | --- | --- | --- | --- | --- | --- | --- |
| Rank | Match Score | Redundant Motif | P-value | log P-value | % of Targets | % of Background | Motif file |
| 1 | 0.766 | C G A T A C T G A C T G C G T A C G T A C T A G C T G A C G T A C T A G C G T A A C T G C A T G C G T A C T G A | 1e-30 | -70.243055 | 2.14% | 0.46% | motif file (matrix) |
